# Xylan-Derived N,S-Codoped Carbon Dots for Hg^2+^ Detection and Reusable Paper-Based Sensing

**DOI:** 10.3390/polym18212753

**Published:** 2026-11-05

**Authors:** Yunqin Qi, Zhouyang Xiang, Qingyi Lin, Zhenhua Hu

**Affiliations:** 1 School of Food Engineering, Ludong University, Yantai 264025, China; 2 State Key Laboratory of Advanced Papermaking & Paper-Based Materials, South China University of Technology, Guangzhou 510640, China

**Keywords:** xylan, N,S-codoped carbon quantum dots, paper-based fluorescence sensing, heavy-metal sensing, hemicellulose valorization

## Abstract

The development of renewable fluorescent nanomaterials for heavy-metal detection provides an opportunity to integrate biomass valorization with functional sensing. Herein, N,S-codoped carbon quantum dots (N,S-CQDs) were prepared by one-step hydrothermal co-carbonization of bagasse-derived xylan, a renewable and hydroxyl-rich hemicellulose selected as the carbon precursor, and L-cysteine. The quasi-spherical N,S-CQDs had a mean diameter of 3.25 ± 0.04 nm and exhibited locally ordered carbon domains and abundant N-, S-, and O-containing surface functionalities. They exhibited excitation-dependent blue fluorescence with optimal excitation/emission wavelengths of 330/420 nm and a relative fluorescence quantum yield of 67.25%. Among the tested metal ions, Hg^2+^ caused the strongest fluorescence quenching, with a concentration-dependent response over 0–1.0 μM and a detection limit of 4.2 nM. Spectroscopic changes and fluorescence recovery after Ethylenediaminetetraacetic acid (EDTA) treatment were consistent with reversible interactions between Hg^2+^ and surface functional groups. Immobilization on filter paper yielded a visually responsive Hg^2+^ sensor with a fluorescence-based detection limit of 6.23 μM and reversible quenching-recovery behavior during repeated Hg^2+^/EDTA treatments. This work integrates renewable xylan valorization, heteroatom-doped carbon dot synthesis, and Hg^2+^-responsive fluorescence, broadening the functional utilization of hemicellulosic resources in portable heavy-metal sensing and providing a design strategy for biomass-derived fluorescent carbon materials for environmental detection.

## 1. Introduction

Selective detection of toxic metal ions using fluorescent carbon nanomaterials is largely governed by their surface chemistry and interfacial interactions with target ions [1]. Accordingly, tailoring the heteroatom composition and surface functional groups of carbon dots to match the coordination characteristics of target ions represents an effective strategy for constructing selective fluorescence detection systems. Hg^2+^ exhibits a strong affinity for sulfur-containing groups and can also interact with certain nitrogen- and oxygen-containing functionalities [2,3]. Introducing S-, N-, and O-containing sites onto the surface of carbon quantum dots (CQDs) can therefore regulate their surface electronic structure and fluorescence behavior while providing potential binding sites for Hg^2+^ [4,5]. On this basis, N,S-codoped carbon quantum dots (N,S-CQDs) have been widely investigated for Hg^2+^ detection [6,7,8,9]. Representative examples include rice-straw-derived N,S-CQDs reported by Nguyen et al. and casein-derived N,S-CQDs prepared by Xu et al., which exhibited a fluorescence quantum yield of 31.8% and a Hg^2+^ detection limit of 6.5 nM [10]. These studies demonstrate that N,S codoping is an established approach for constructing Hg^2+^-responsive carbon dots.

The interfacial properties of N,S-CQDs are governed not only by the heteroatom source but also by the structure of the carbon precursor and the hydrothermal carbonization process [11]. Natural polysaccharides are attractive renewable precursors for carbon dot synthesis because their hydroxyl-rich structures can undergo dehydration, condensation, and carbonization under hydrothermal conditions, giving rise to nanoscale carbon domains together with abundant oxygen-containing surface functionalities [12,13,14]. Co-carbonization of polysaccharides with N/S-containing precursors enables simultaneous carbon-core formation and heteroatom incorporation, providing a direct route for regulating the fluorescence and ion-responsive properties of carbon dots. Beyond material synthesis, the use of polysaccharide precursors also links functional carbon-nanomaterial construction with the valorization of renewable biomass resources [15].

Xylan is a major hemicellulosic component of lignocellulosic biomass, and its hydroxyl-rich polysaccharide structure makes it a renewable precursor for hydrothermal carbonization [16]. Previous studies have demonstrated the potential of xylan-derived carbon dots in fluorescence-related applications. For example, Ma et al. developed xylan-derived carbon dots with tunable fluorescence for white light-emitting diodes [17], while Feng et al. prepared xylan-based fluorescent carbon dots for Cu^2+^ detection [18]. More directly related to Hg^2+^ sensing, Zhang et al. developed xylan-based ratiometric fluorescent carbon dots through subsequent amidation, achieving a detection limit of 1.7 nM. These carbon dots were further incorporated into delignified wood for Hg^2+^ detection and adsorption [19]. These studies demonstrate the versatility of xylan as a precursor for functional fluorescent carbon materials. In contrast to the post-synthetic functionalization strategy reported for xylan-based Hg^2+^ sensing, the present study employs one-step hydrothermal co-carbonization of bagasse-derived xylan and L-cysteine to prepare N,S-CQDs without a separate post-synthetic amidation step. The resulting carbon dots are further immobilized on filter paper to investigate Hg^2+^-responsive fluorescence and EDTA-assisted signal recovery.

Beyond fluorescence detection in solution, immobilization of carbon dots on cellulosic and paper-based substrates provides a straightforward route toward portable and visually readable sensing formats [20,21,22,23,24]. Carbon dots incorporated into cellulose-based matrices can retain useful fluorescence characteristics in the solid-state, supporting their further development as processable fluorescent sensing materials [25]. In particular, paper-based carbon dot systems have been reported for Hg^2+^ detection, where fluorescence changes can be directly visualized after exposure to the target ion [26,27]. For systems governed by reversible interfacial interactions, competitive complexing agents can additionally alter the interaction between Hg^2+^ and the carbon dot surface. The strong complexation of Hg^2+^ by EDTA provides a basis for fluorescence recovery and repeated use of the sensing material [28,29]. Accordingly, extending the Hg^2+^ response of N,S-CQDs from solution to a paper-based platform provides a simple strategy for evaluating their visual response, reversibility, and suitability for reusable fluorescence sensing.

In this work, N,S-CQDs were prepared through one-step hydrothermal co-carbonization using xylan as a renewable polysaccharide precursor and L-cysteine as an N/S-rich co-precursor. Their nanoscale morphology, carbon structure, surface chemical composition, and fluorescence properties were systematically characterized. The selectivity and concentration-dependent fluorescence response of the N,S-CQDs toward Hg^2+^ were evaluated, and the possible response process was examined on the basis of the observed spectroscopic changes. The N,S-CQDs were further immobilized on filter paper to construct a visual and reusable paper-based fluorescence sensing platform, with reversibility evaluated through alternating Hg^2+^/EDTA treatments. Their compatibility with paper-based processing was also demonstrated through fluorescent writing and pattern printing. Compared with previously reported xylan-derived and N,S-codoped carbon dot systems, this work focuses on integrating renewable xylan utilization, one-step heteroatom-containing carbon dot preparation, and reversible paper-based Hg^2+^ fluorescence response within a single material platform. These findings provide an experimental basis for the further development of hemicellulose-derived fluorescent materials and paper-based sensing systems.

## 2. Experimental Section

### 2.1. Materials and Reagents

Bagasse was purchased from Yunnan Yunjing Forestry and Pulp Co., Ltd., China, and used as the primary biomass feedstock. L-cysteine, hydrochloric acid, EDTA, sodium hydroxide, and the metal salts used in the experiments were purchased from Shanghai Macklin Biochemical Co., Ltd. (Shanghai, China). L-cysteine had a purity of 90%, while hydrochloric acid and sodium hydroxide were of analytical grade. Deionized water (18.2 MΩ·cm) was used for the preparation of all aqueous solutions.

The metal salts used for the selectivity tests included mercury(II) sulfate, nickel(II) sulfate hexahydrate, magnesium sulfate, lithium sulfate, silver nitrate, anhydrous calcium sulfate, anhydrous iron(III) chloride, anhydrous copper(II) chloride, anhydrous zinc sulfate, potassium chloride, barium chloride, sodium chloride, iron(II) sulfate, cadmium nitrate tetrahydrate, chromium(III) nitrate nonahydrate, manganese sulfate, cobalt sulfate, aluminum sulfate, and lead nitrate. Unless otherwise specified, all reagents were used as received without further purification.

### 2.2. Extraction of Xylan and Preparation of N,S-CQDs

Xylan was isolated from sugarcane bagasse using an established alkaline extraction procedure previously reported by our research group, with modifications to the extraction and purification conditions [30,31]. The procedure involved ethanol extraction, delignification, alkaline extraction, ethanol precipitation, and dialysis purification. Bagasse was first subjected to Soxhlet extraction with 95% ethanol for 6 h to remove lipids, waxes, and low-molecular-weight soluble components. The extracted bagasse was dried overnight at 60 °C before further treatment.

The dried bagasse was delignified using an acidified NaClO_2_ system. The sample was dispersed in a 4 wt% NaClO_2_ solution at a solid-to-liquid ratio of 1:20 and treated at 75 °C and pH 3.9–4.0 for 1 h. After the reaction, the solid residue was collected by vacuum filtration and subjected to a second bleaching treatment with a 2 wt% NaClO_2_ solution at the same solid-to-liquid ratio. The resulting solid was washed with deionized water until the washings were neutral, further washed with absolute ethanol, and then freeze-dried using a freeze dryer (FDU-2110, Rikakikai Co., Ltd., Tokyo, Japan) to obtain holocellulose.

The holocellulose was dispersed in a 2 wt% NaOH solution at a solid-to-liquid ratio of 1:20 and treated at 90 °C for 3 h to extract the hemicellulosic fraction [32]. After the reaction, the insoluble residue was removed by vacuum filtration, and the alkaline extract was collected. The pH of the filtrate was then adjusted to approximately 5.5 with glacial acetic acid, followed by the addition of three volumes of absolute ethanol to induce xylan precipitation [33]. After standing overnight, the precipitate was collected by centrifugation using a G-16 centrifuge (Sartorius Lab Instruments GmbH & Co. KG, Göttingen, Germany) and washed three times with 70% ethanol. The resulting precipitate was transferred into dialysis tubing with a molecular weight cut-off (MWCO) of 3500 Da and dialyzed against deionized water for 1 week, followed by freeze-drying to obtain crude xylan. For further purification, the crude xylan was redissolved in a 2 wt% NaOH solution and reprecipitated with excess absolute ethanol. The precipitate was washed with absolute ethanol, dialyzed again against deionized water for 1 week using 3500 Da MWCO dialysis tubing, and freeze-dried to obtain purified xylan.

N,S-CQDs were prepared by hydrothermal co-carbonization of xylan and L-cysteine. Xylan (0.1 g) and L-cysteine (0.1 g) were dissolved in 25 mL of ultrapure water and thoroughly stirred to form a homogeneous precursor solution. The resulting solution was transferred into a 50 mL hydrothermal autoclave and heated at 200 °C for 12 h. After the reaction, the mixture was allowed to cool naturally to room temperature. The resulting product was filtered through a 0.22 μm membrane and centrifuged at 10,000 rpm for 10 min using a G-16 centrifuge (Sartorius, Germany). The supernatant was collected, subjected to an extraction treatment, and then dialyzed for 1 week using a cellulose dialysis membrane with an MWCO of 500 Da. The dialysate was filtered again through a 0.22 μm membrane and stored at 4 °C until further use. The synthetic route for the N,S-CQDs is illustrated in Figure S1. The mass concentration of the N,S-CQD dispersion was determined gravimetrically by drying 3 mL of the dispersion to constant weight in an oven at 60 °C and calculating the concentration from the mass of the residual solids. The mass concentration of the N,S-CQD stock dispersion was determined to be 2.53 mg/mL.

### 2.3. Preparation of the Paper-Based Sensor and Fluorescent Ink

The N,S-CQD paper-based sensor was prepared by an immersion method. Filter paper was cut into rectangular pieces measuring 1.5 cm × 2.0 cm and immersed in the N,S-CQD dispersion for 30 min to allow sufficient adsorption of the N,S-CQDs onto the paper fibers. The filter-paper pieces were then removed and dried naturally at room temperature. To increase the loading of N,S-CQDs on the paper substrate, the immersion-drying process was repeated five times to obtain the N,S-CQD paper-based sensors.

For fluorescent-ink applications, N,S-CQD dispersions were directly used as writing and printing inks. Specifically, a cotton swab was dipped into N,S-CQD dispersions of different relative concentrations and used to write on filter paper. In parallel, a stamp was wetted with the N,S-CQD dispersion and pressed onto the filter paper to produce printed patterns. After air-drying at room temperature, the resulting text and patterns were observed under visible light and UV irradiation.

### 2.4. Characterization of N,S-CQDs

The structural characteristics, surface chemical composition, and optical properties of the N,S-CQDs were investigated using Fourier transform infrared spectroscopy (FT-IR), transmission electron microscopy (TEM), atomic force microscopy (AFM), X-ray diffraction (XRD), X-ray photoelectron spectroscopy (XPS), UV-Vis absorption spectroscopy, and fluorescence spectroscopy. FT-IR spectra of xylan and N,S-CQDs were recorded using a Tensor 27 spectrometer (Bruker Optik GmbH, Ettlingen, Germany) over the range of 4000–400 cm^−1^ at a resolution of 4 cm^−1^ to examine changes in functional groups before and after the hydrothermal reaction [34]. An appropriately diluted N,S-CQD dispersion was deposited onto an ultrathin carbon-coated grid and dried at room temperature before TEM and high-resolution TEM (HRTEM) observations using a JEM-2100F transmission electron microscope (JEOL, Tokyo, Japan). A total of 83 particle diameters were included in the TEM-based particle-size analysis. The measured diameters in pixels were converted into nanometers using a calibration factor of 0.4239245 nm/pixel. The particle-size results were expressed as the mean ± sample standard deviation (SD). To obtain height information, the diluted N,S-CQD dispersion was deposited onto freshly cleaved mica and dried before characterization using a Dimension Icon atomic force microscope (Bruker, Germany). XRD analysis was performed using an X’Pert Powder diffractometer (PANalytical, Almelo, The Netherlands) over a 2θ range of 5–60° at a scanning rate of 2°/min [35]. XPS measurements were performed using a K-Alpha spectrometer (Waltham, MA, USA). Survey spectra and high-resolution C 1s, O 1s, N 1s, and S 2p spectra were collected to investigate the elemental composition and surface chemical environments of the N,S-CQDs. The high-resolution spectra were analyzed by curve fitting to resolve overlapping contributions associated with different chemical environments. The component assignments were evaluated according to their binding-energy positions and relevant XPS reference data, following established principles of XPS peak fitting. The relative contribution of each fitted component was determined from its integrated area within the corresponding core-level spectrum. The surface atomic percentages of C, N, O, and S were obtained from the Atomic % values in the XPS quantitative peak table.

The optical properties of the N,S-CQDs were characterized by steady-state fluorescence spectroscopy and three-dimensional excitation–emission spectroscopy. Steady-state fluorescence spectra were recorded using a FluoroMax-4 fluorescence spectrometer (HORIBA Scientific, Edison, NJ, USA), with both the excitation and emission slit widths set to 10 nm. Emission spectra were recorded at different excitation wavelengths to investigate the excitation-dependent fluorescence behavior of the N,S-CQDs. Three-dimensional excitation–emission spectra were recorded to further characterize the excitation–emission distribution, while CIE chromaticity coordinates were used to describe the fluorescence color. To evaluate the fluorescence stability of the N,S-CQDs, changes in fluorescence intensity were monitored over 0–24 h under identical measurement conditions. For the pH-response experiments, the N,S-CQDs were dispersed in solutions with pH values ranging from 1 to 14, equilibrated for 15 min, and then measured at an excitation wavelength of 330 nm.

The relative fluorescence quantum yield of the N,S-CQDs was evaluated using a comparative method. Quinine sulfate dissolved in 0.1 M H_2_SO_4_ was used as the reference standard, with a reported fluorescence quantum yield of 54% [36]. The excitation wavelength was set to 330 nm, corresponding to the optimal excitation wavelength of the N,S-CQDs. N,S-CQD aqueous dispersions were prepared at different concentrations, and their absorbance values at 330 nm were measured using UV–Vis spectroscopy. The corresponding fluorescence emission spectra were recorded, and the integrated fluorescence emission intensities were calculated over the selected emission wavelength range [37].

Five absorbance–integrated fluorescence intensity data pairs were analyzed for the N,S-CQDs, with absorbance values ranging from 0.020 to 0.080. The corresponding quinine sulfate reference calibration data were obtained from published literature. Linear regression of integrated fluorescence intensity against absorbance was performed to determine the slopes for the N,S-CQDs (Grad_x_) and the quinine sulfate reference (Grad_st_). The absorbance values, integrated fluorescence intensities, and regression parameters are summarized in Table S1. The relative fluorescence quantum yield was subsequently calculated according to Equation (1) [16,38]:

(1) $$Q_{X}=Q_{st}\frac{{\mathrm{Grad}}_{x}\eta_{x}^{2}}{{\mathrm{Grad}}_{\mathrm{st}}\eta_{\mathrm{st}}^{2}}$$

Here, *Q_x_* and *Q_st_* are the fluorescence quantum yields of the N,S-CQDs and quinine sulfate standard, respectively. *Grad_x_* and *Grad_st_* are the slopes obtained from the linear plots of integrated fluorescence intensity versus absorbance for the sample and reference, respectively. *η_x_* and *η_st_* are the refractive indices of the corresponding solvents. The subscripts *x* and *st* denote the sample and reference standard, respectively.

Fluorescence decay curves and lifetime parameters of the N,S-CQDs were measured using an FLS1000 fluorescence spectrometer (Edinburgh Instruments, Livingston, UK). The fluorescence decay curves were fitted using a biexponential decay model according to Equation (2) [39]:

(2) $$Y\;=A_{1}\exp\left(\frac{-t}{\tau_{1}}\right)+A_{2}\exp\left(\frac{-t}{\tau_{2}}\right)$$

The average fluorescence lifetime of the N,S-CQDs was calculated according to Equation (3) [40]:

(3) $$\bar{\tau }=\frac{A_{1}\tau_{1}^{2}+A_{2}\tau_{2}^{2}}{A_{1}\tau_{1}+A_{2}\tau_{2}}$$

Here, *Y* is the fluorescence intensity. *A*_1_ and *A*_2_ are the relative amplitudes of the two lifetime components. *τ*_1_ and *τ*_2_ are the corresponding fluorescence lifetimes, and *τ* is the average fluorescence lifetime.

### 2.5. Fluorescence Detection of Hg^2+^ and Selectivity Tests

The fluorescence response of the N,S-CQDs toward Hg^2+^ was evaluated through metal ion selectivity tests and Hg^2+^ concentration-dependent measurements. Aqueous solutions (2 μM) of Ag^+^, Na^+^, Li^+^, K^+^, Hg^2+^, Cu^2+^, Fe^3+^, Fe^2+^, Ba^2+^, Pb^2+^, Zn^2+^, Ca^2+^, Cd^2+^, Ni^2+^, Cr^3+^, Mg^2+^, Co^2+^, Mn^2+^, and Al^3+^ were prepared for the selectivity tests. The N,S-CQD dispersion, diluted by a factor of 1.5, was mixed with each metal ion solution at a 1:1 volume ratio and incubated for 15 min before the fluorescence emission spectra were recorded using a FluoroMax-4 fluorescence spectrometer (HORIBA, USA). Selectivity toward different metal ions was evaluated by comparing the fluorescence intensity of the N,S-CQDs before and after addition of the corresponding ions. Unless otherwise specified, the Hg^2+^ concentrations reported in this section refer to the final concentrations in the detection system after mixing with the N,S-CQD working dispersion, and the final concentration of each metal ion in the selectivity tests was 1.0 μM.

The fluorescence response of the N,S-CQDs was measured over a range of Hg^2+^ concentrations. A 150 μL aliquot of the N,S-CQD working dispersion was mixed with 150 μL of an Hg^2+^ working solution to obtain a total volume of 300 μL. The final Hg^2+^ concentration in the detection system ranged from 0 to 1.0 μM. After incubation for 15 min, the fluorescence emission spectra were recorded. The fluorescence intensity in the absence of Hg^2+^ was defined as I_0_, while that in the presence of Hg^2+^ was defined as I, and the relative fluorescence intensity I/I_0_ was plotted against the Hg^2+^ concentration to establish the calibration relationship. The limit of detection (LOD) was calculated according to Equation (4) [41]:

(4) $$\mathrm{LOD}\;=\;\frac{3\sigma }{\left|k\right|}$$

Here, *σ* is the standard deviation of the relative fluorescence response obtained from replicate blank measurements, and *k* is the slope of the calibration curve. Each concentration was measured in triplicate, and the results are expressed as the mean ± standard deviation.

To examine the absorption changes induced by Hg^2+^, UV-Vis absorption spectra of the N,S-CQDs, N,S-CQDs/Hg^2+^, and N,S-CQDs/Hg^2+^/EDTA systems were recorded using a UV-2600i UV-Vis spectrophotometer (Shimadzu Instruments (Suzhou) Co., Ltd., Suzhou, China).

### 2.6. Paper-Based Detection and Reusability Tests

The detection performance of the N,S-CQD paper-based sensor was evaluated by solid-state fluorescence measurements and visual drop-casting tests. Solid-state fluorescence spectra were recorded using a FluoroMax-4 fluorescence spectrometer (HORIBA, USA) equipped with a solid-sample accessory. During measurement, the paper sample was mounted on the solid-sample holder with the N,S-CQD-loaded surface facing the excitation beam, and the fluorescence emission spectra were recorded using a front-face detection geometry. Unmodified filter paper was used as the background control to correct for scattering and background fluorescence from the paper substrate. Each concentration was tested in triplicate, and the results are expressed as the mean ± standard deviation.

For the selectivity tests, 1 mL of each 0.1 mM metal ion solution was uniformly applied to the N,S-CQD paper-based sensor in several portions, and the fluorescence spots and solid-state fluorescence intensities after drying were compared to evaluate the visual response toward Hg^2+^ [42]. The fluorescence response of the paper-based sensor toward different Hg^2+^ concentrations was further investigated, with I/I_0_ plotted against the Hg^2+^ concentration to establish a calibration relationship, and the LOD was calculated according to Equation (4).

The reusability of the paper-based sensor was evaluated by alternating Hg^2+^ and EDTA treatments. After fluorescence quenching by Hg^2+^ treatment, the paper-based sensor was immersed in a saturated EDTA solution for 30 min to complex Hg^2+^ retained in the paper substrate. The sample was then removed, gently rinsed with a small amount of ultrapure water, dried at room temperature, and reused for Hg^2+^ detection. The quenching-recovery process was repeated to evaluate the reversibility and reusability of the paper-based sensor.

## 3. Results and Discussion

### 3.1. Physicochemical Characterization of N,S-CQDs

**Figure 1:**
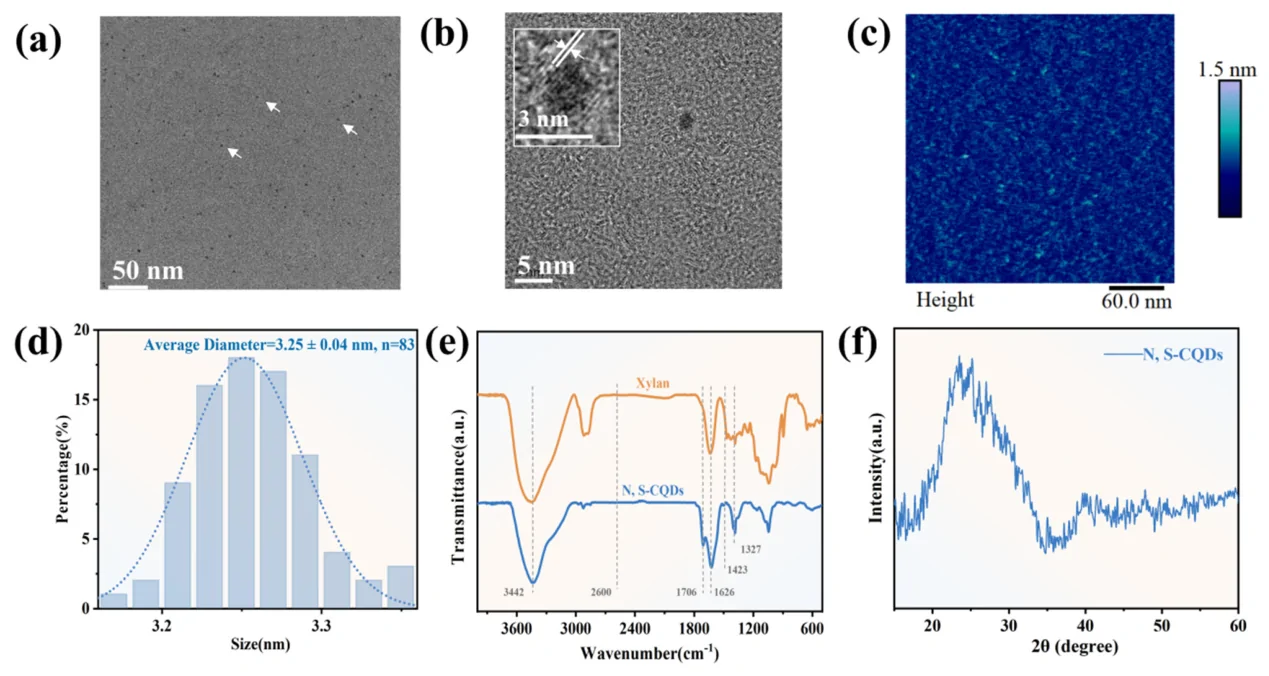
(**a**) TEM image, (**b**) HRTEM image, (**c**) AFM image of N,S-CQDs, (**d**) particle-size distribution histogram, (**e**) FT-IR spectra of xylan and N,S-CQDs and (**f**) XRD pattern of N,S-CQDs.

The morphology and size characteristics of the N,S-CQDs are shown in Figure 1. The TEM image shows that the N,S-CQDs are quasi-spherical nanoparticles that are relatively uniformly dispersed within the observed area, with no obvious large-scale aggregation (Figure 1a). Particle-size analysis shows that the particle diameters are mainly distributed between 3 and 4 nm, with a mean diameter of 3.25 ± 0.04 nm (mean ± SD, number of particles = 83), indicating a relatively narrow particle-size distribution (Figure 1d), indicating a small nanoscale size and a relatively narrow size distribution.

The HRTEM image (Figure 1b) reveals distinguishable lattice fringes within the N,S-CQDs, which can be more clearly observed in the enlarged inset. The measured lattice spacing is approximately 0.21 nm, consistent with the (100) crystallographic plane of graphitic carbon reported in previous studies [35,43]. This spacing is associated with the in-plane ordering of graphitic carbon structures, suggesting the presence of locally ordered carbon domains within the N,S-CQDs. The AFM image further shows that the particles are relatively uniformly distributed on the mica substrate, with a height variation of approximately 1 nm across the scanned area (Figure 1c). Taken together, the TEM, HRTEM, and AFM results confirm the nanoscale dimensions of the N,S-CQDs and indicate the presence of a certain degree of local carbon ordering.

The FT-IR spectra of the extracted xylan and the resulting N,S-CQDs are presented in Figure 1e, Table S2. The extracted xylan exhibits a broad absorption band near 3442 cm^−1^, which is mainly attributed to O-H stretching vibrations. The absorption bands in the 1000–1170 cm^−1^ region are mainly associated with C-O and C-O-C stretching vibrations of the polysaccharide structure, whereas the band near 898 cm^−1^ is consistent with β-glycosidic linkages in the xylan backbone [44]. Following hydrothermal co-carbonization, noticeable changes are observed in the FT-IR spectrum of the resulting N,S-CQDs. The broad absorption band near 3442 cm^−1^ may arise from overlapping O-H and N-H stretching vibrations. The absorption band near 1706 cm^−1^ is consistent with C=O stretching vibrations, while the feature near 1626 cm^−1^ may involve contributions from conjugated C=C stretching and other overlapping vibrational modes. The absorption features near 1423 and 1327 cm^−1^ may be associated with overlapping C-N, C-O, and related deformation vibrations and therefore cannot be attributed exclusively to nitrogen-containing functional groups. A weak absorption feature is also observed near 2600 cm^−1^ [45]. Although this region is consistent with the characteristic frequency range of S-H stretching vibrations reported in related carbon dot systems, the available FT-IR evidence is insufficient for an unambiguous assignment to surface thiol groups [45,46]. Overall, the observed FT-IR spectral changes are consistent with chemical transformations of the precursor system during hydrothermal treatment and changes in the functional-group environments of the resulting N,S-CQDs. Nevertheless, the precise chemical structures of individual surface functionalities cannot be established solely from these vibrational features.

The XRD pattern of the N,S-CQDs is presented in Figure 1f, showing a broad diffraction peak at approximately 2θ = 25–26°, which is commonly associated with the (002) reflection of disordered graphitic carbon [35,43]. The broad diffraction feature indicates limited long-range structural ordering, suggesting that the N,S-CQDs predominantly possess a poorly crystalline carbon structure. This observation is consistent with the HRTEM results, where lattice fringes corresponding to the (100) plane of graphitic carbon are observed within localized regions. Although the two characterization techniques probe different aspects of carbon ordering, their combined results suggest that the N,S-CQDs consist mainly of disordered carbon with locally ordered graphitic domains.

XPS was employed to investigate the surface elemental composition and chemical states of the N,S-CQDs, as shown in Figure S2. The survey spectrum exhibits characteristic C 1s, O 1s, N 1s, and S 2p signals (Figure S2a), confirming the presence of carbon, oxygen, nitrogen, and sulfur in the resulting N,S-CQDs. Quantitative XPS analysis revealed surface atomic percentages of 60.51% C, 32.75% O, 3.44% N, and 3.30% S, further supporting the incorporation of nitrogen- and sulfur-containing species into the N,S-CQDs. The incorporation of nitrogen and sulfur is consistent with the participation of L-cysteine during hydrothermal synthesis. The high-resolution C 1s spectrum (Figure S2b) was deconvoluted into four components assigned to C-C/C=C, C-O, C=O, and O-C=O, accounting for 53.5%, 28.9%, 10.2%, and 7.4% of the total fitted C 1s peak area, respectively. These assignments are consistent with the characteristic carbon bonding environments reported in previous XPS studies [47]. The dominant C-C/C=C contribution indicates the formation of a carbon-rich framework, while the remaining components reveal the presence of oxygen-containing functional groups. Similarly, the O 1s spectrum (Figure S2c) was resolved into three components associated with C-O/O-H, O-C=O, and C=O environments, contributing 61.7%, 32.1%, and 6.2%, respectively.

The high-resolution N 1s spectrum (Figure S2d) was fitted with four components associated with amino N, graphitic N, pyrrolic N, and pyridinic N, with relative contributions of 19.9%, 19.8%, 45.0%, and 15.3%, respectively. Among these components, pyrrolic N exhibits the largest relative contribution. The coexistence of different nitrogen-containing structures is consistent with previous studies of N,S-codoped carbon quantum dots [48,49] and supports the incorporation of nitrogen-containing species during hydrothermal carbonization. The S 2p spectrum (Figure S2e) exhibits two fitted contributions, labeled as -SH-related and -SO_3_H-related sulfur environments, with relative peak-area contributions of 64.5% and 35.5%, respectively. These features indicate the presence of sulfur-containing chemical environments in the resulting N,S-CQDs. The interpretation of the S 2p spectrum also takes into account the characteristic spin–orbit structure discussed in established XPS fitting guidelines [50,51]. Overall, the XPS results demonstrate the successful introduction of nitrogen- and sulfur-containing structures into the carbon dot system and reveal the coexistence of various oxygen-, nitrogen-, and sulfur-containing surface functionalities. These chemical features contribute to the surface characteristics of the N,S-CQDs and provide potential interaction sites for Hg^2+^.

### 3.2. Optical Properties of N,S-CQDs

**Figure 2:**
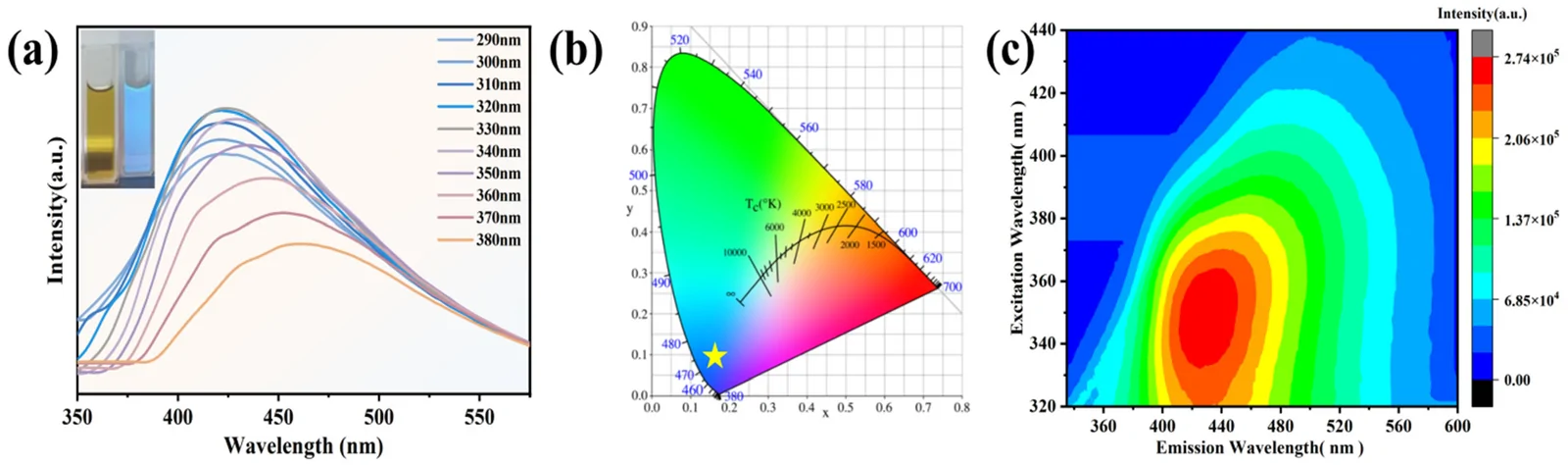
(**a**) Fluorescence emission spectra of N,S-CQDs at different excitation wavelengths; the inset shows photographs of the N,S-CQD dispersion under visible light and UV irradiation. (**b**) CIE chromaticity diagram (The **yellow star** marks the calculated color coordinates of the sample) and (**c**) three-dimensional excitation–emission spectrum of N,S-CQDs.

The fluorescence emission characteristics, CIE chromaticity coordinates, and three-dimensional excitation–emission spectrum of the N,S-CQDs are presented in Figure 2. As shown in Figure 2a, the N,S-CQDs exhibit pronounced blue fluorescence emission under different excitation wavelengths. As the excitation wavelength increases from 290 to 380 nm, the emission maximum gradually shifts to longer wavelengths, while the fluorescence intensity first increases and then decreases, demonstrating clear excitation-dependent emission behavior. The optimal excitation wavelength is approximately 330 nm, corresponding to a maximum emission wavelength of approximately 420 nm. The inset shows that the N,S-CQD dispersion appears pale yellow under visible light and emits bright blue fluorescence under UV irradiation, consistent with its fluorescence emission characteristics. The excitation-dependent emission of the N,S-CQDs may be associated with differences in the surface chemical environment and the presence of multiple surface- and defect-state emissive centers [52,53,54]. Emissive centers with different energy levels may be selectively excited at different excitation wavelengths, resulting in changes in both emission wavelength and fluorescence intensity. The CIE chromaticity coordinates are located in the blue region (Figure 2b), further confirming the blue fluorescence emission of the N,S-CQDs. The three-dimensional excitation–emission spectrum shows that the N,S-CQDs exhibit fluorescence over a relatively broad excitation and emission range, with a continuous excitation–emission distribution (Figure 2c). This result is consistent with the excitation-dependent shift in the emission maximum and further suggests that the fluorescence behavior of the N,S-CQDs may involve multiple surface- or defect-state emissive centers.

To evaluate the short-term fluorescence stability, the fluorescence intensity of the N,S-CQDs was monitored as a function of storage time under fixed excitation conditions (Figure S3a) [55]. The fluorescence intensity of the N,S-CQDs is strongly dependent on pH (Figure S3b). The N,S-CQDs exhibit stronger fluorescence under mildly acidic to near-neutral conditions, with the maximum intensity observed at approximately pH 6, whereas strongly acidic or alkaline conditions lead to a decrease in fluorescence intensity. This behavior may be associated with protonation and deprotonation of oxygen-, nitrogen-, and sulfur-containing surface functional groups [56]. Changes in solution acidity can alter the surface charge state and electronic environment of the surface states, thereby affecting the fluorescence emission process.

The fluorescence decay curve of the N,S-CQDs is shown in Figure S3c and can be fitted using a biexponential model, indicating the presence of multiple decay components [57]. The fitted short- and long-lifetime components are approximately 1.43 and 7.71 ns, respectively, with an average fluorescence lifetime of approximately 6.35 ns. The relative fluorescence quantum yield is 67.25% (Table S3). Combined with the excitation-dependent emission and surface-chemistry results, the fluorescence behavior of the N,S-CQDs may reflect from contributions from both the carbon-core structure and surface states. In addition, the N,S-CQD dispersions display pronounced blue fluorescence under UV irradiation (Figure S3d), consistent with the fluorescence emission spectra and CIE chromaticity analysis. Overall, the N,S-CQDs exhibit good short-term fluorescence stability, a pronounced pH-dependent response, a relatively high fluorescence quantum yield, and readily observable fluorescence, providing a basis for subsequent fluorescence detection experiments.

### 3.3. Fluorescence Detection of Hg^2+^ and Possible Response Mechanism

**Figure 3:**
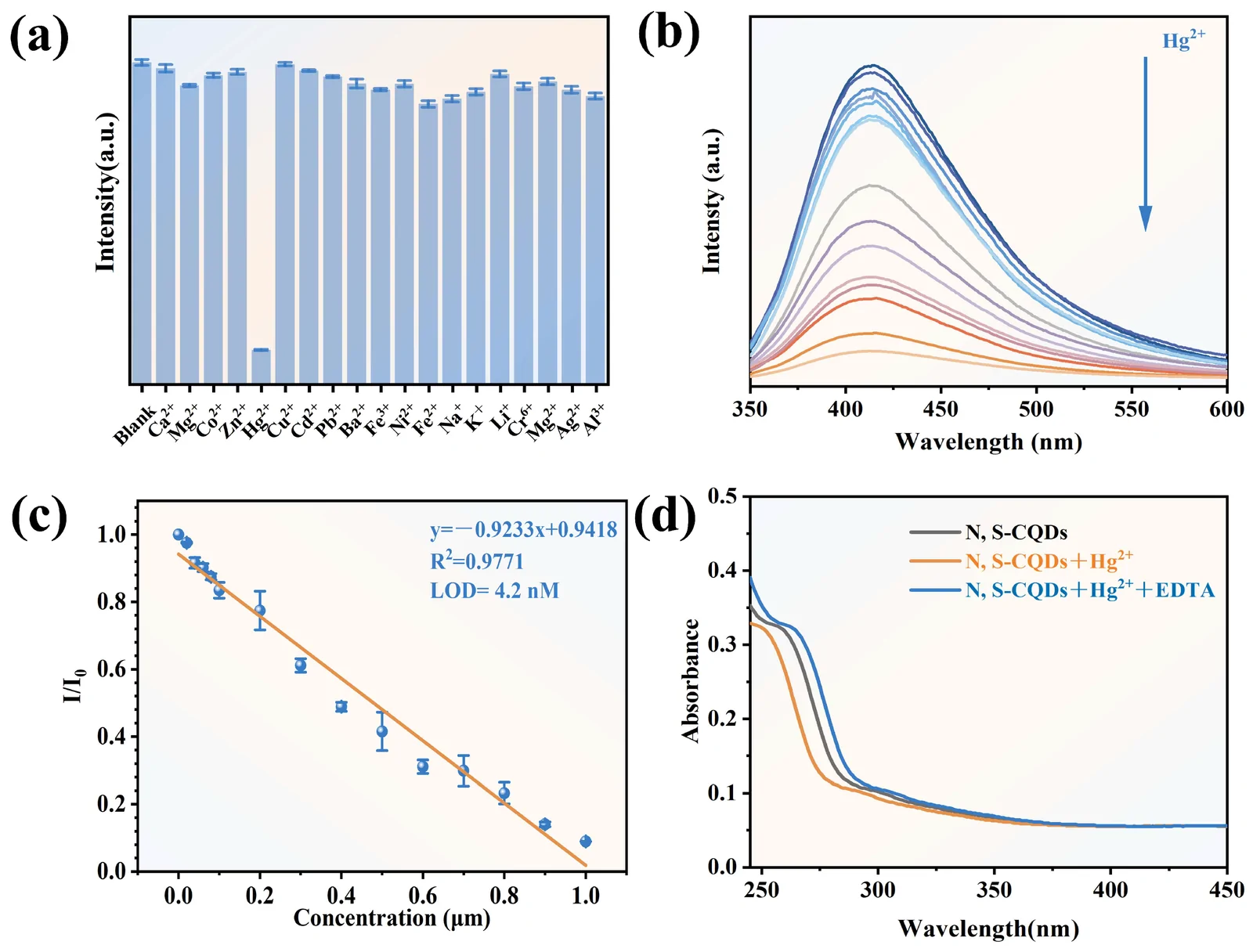
(**a**) Effects of different metal ions on the fluorescence intensity of N,S-CQDs; (**b**) fluorescence emission spectra of N,S-CQDs at different Hg^2+^ concentrations (From top to bottom, the curves correspond to Hg^2+^ concentrations of 0, 0.02, 0.04, 0.06, 0.08, 0.1, 0.2, 0.3, 0.4, 0.5, 0.6, 0.7, 0.8, 0.9, and 1 μM, respectively); (**c**) relationship between *I*/*I*_0_ and Hg^2+^ concentration; and (**d**) UV-Vis absorption spectra of N,S-CQDs, N,S-CQDs/Hg^2+^, and N,S-CQDs/Hg^2+^/EDTA systems.

To evaluate the selectivity of the N,S-CQDs toward metal ions, the effects of various metal cations on their fluorescence intensity were examined, as shown in Figure 3. Under identical test conditions, Ag^+^, Na^+^, Li^+^, K^+^, Cu^2+^, Fe^3+^, Fe^2+^, Ba^2+^, Pb^2+^, Zn^2+^, Ca^2+^, Cd^2+^, Ni^2+^, Cr^3+^, Mg^2+^, Co^2+^, Mn^2+^, and Al^3+^ caused relatively small changes in fluorescence intensity compared with Hg^2+^. In contrast, the addition of Hg^2+^ resulted in a pronounced decrease in fluorescence intensity, indicating a marked fluorescence quenching response. These results indicate that the N,S-CQDs exhibit a preferential fluorescence quenching response toward Hg^2+^ compared with the other individually tested metal ions under identical experimental conditions. As indicated by the FT-IR and XPS analyses, the N,S-CQD surface contains abundant functional groups. Among these groups, sulfur- and nitrogen-containing functionalities may provide potential interaction sites for Hg^2+^, and the resulting surface interactions may alter the surface-state environment and the associated fluorescence emission process, leading to fluorescence quenching [44]. The stronger interaction of Hg^2+^ with surface binding sites of the N,S-CQDs compared with the other tested metal ions may contribute to the observed selective response.

The effect of Hg^2+^ concentration on the fluorescence emission of the N,S-CQDs was further investigated. As shown in Figure 3b, the fluorescence intensity decreases progressively with increasing Hg^2+^ concentration, while the overall spectral profile remains largely unchanged, indicating that Hg^2+^ mainly affects the emission intensity. The relative fluorescence intensity I/I_0_ was plotted against the Hg^2+^ concentration, where I_0_ and I denote the fluorescence intensities in the absence and presence of Hg^2+^, respectively. Within the concentration range of 0–1.0 μM, an approximately linear relationship was obtained:

*y* = −0.9233*C_Hg_*^2+^ + 0.9418

The corresponding coefficient of determination was *R*^2^ = 0.9771. Based on the *LOD =* 3*σ/|k|* criterion, the limit of detection was calculated to be 4.2 nM (Figure 3c), indicating sensitivity toward low concentrations of Hg^2+^.

To further investigate the possible fluorescence quenching mechanism, the UV-Vis absorption characteristics of the N,S-CQDs before and after Hg^2+^ addition were compared (Figure 3d). The observed spectral changes suggest that Hg^2+^ affects the local chemical or electronic environment of the N,S-CQDs. As revealed by FT-IR and XPS analyses, the N,S-CQDs contain sulfur-, nitrogen-, and oxygen-containing surface functional groups that may serve as interaction sites for Hg^2+^. According to the hard–soft acid-base principle, Hg^2+^, as a soft Lewis acid, exhibits a strong affinity for sulfur-containing coordination sites, while nitrogen- and oxygen-containing groups may also participate in surface interactions. Such interactions may alter the electronic distribution and surface-related emissive states of the N,S-CQDs, potentially promoting nonradiative relaxation and charge-transfer processes [58]. This interpretation is consistent with the concentration-dependent fluorescence quenching observed in Figure 3b, where the fluorescence intensity decreases progressively with increasing Hg^2+^ concentration while the overall emission spectral profile remains largely unchanged. Therefore, the fluorescence quenching behavior is proposed to arise primarily from Hg^2+^ interactions with surface functional groups and the resulting modulation of surface-state-related emission processes.

### 3.4. Paper-Based Visual Detection and Reversible Response Toward Hg^2+^

**Figure 4:**
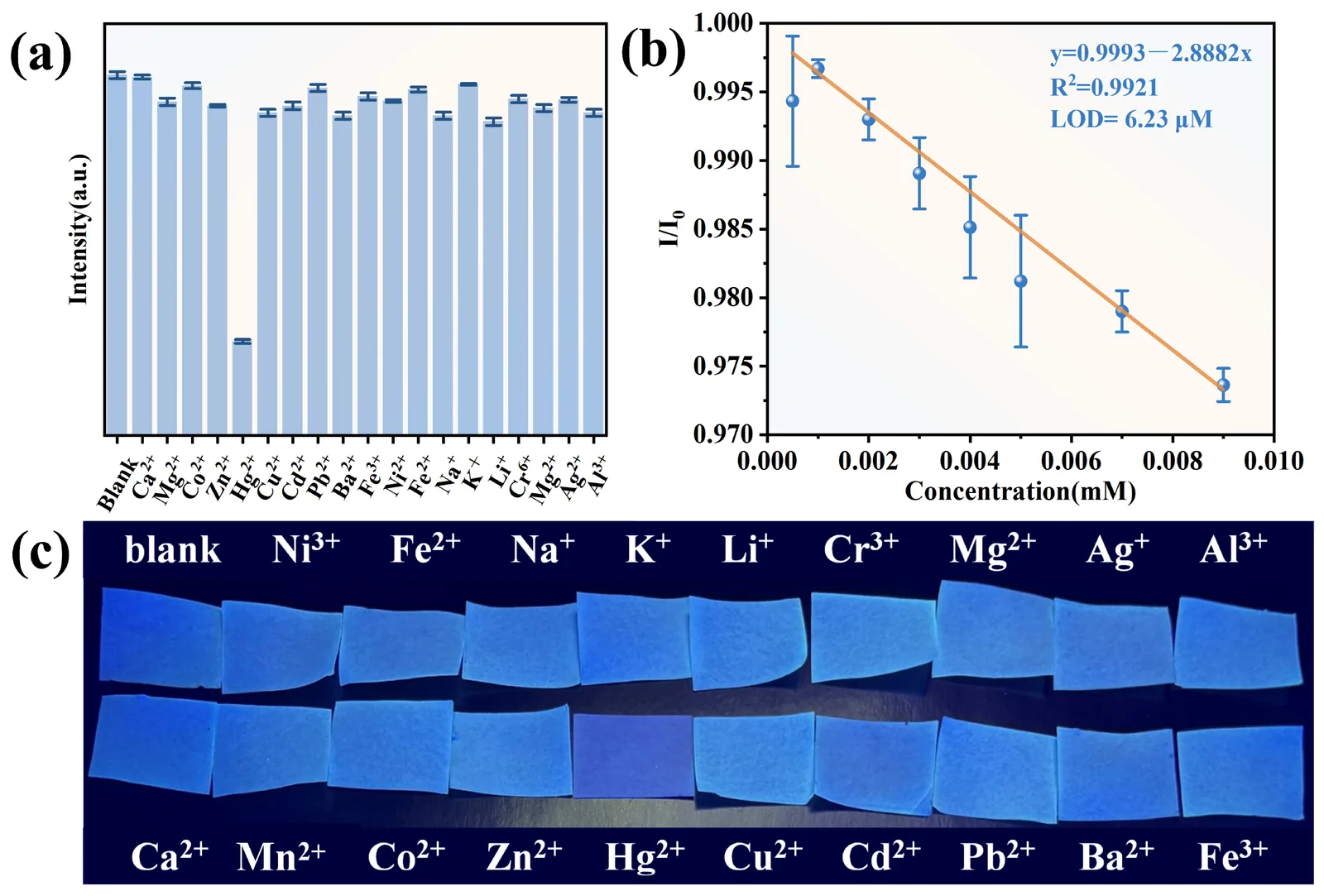
Detection performance of the N,S-CQD paper-based sensor toward Hg^2+^: (**a**) effects of different metal ions on fluorescence intensity; (**b**) relationship between I/I0 and Hg^2+^ concentration; and (**c**) fluorescence photographs of paper-based sensors treated with different metal ions under UV irradiation.

The metal ion selectivity and Hg^2+^ response of the N,S-CQD-loaded filter paper are shown in Figure 4. Under identical test conditions, most metal ions caused only minor changes in the fluorescence intensity of the paper-based sensor, whereas treatment with Hg^2+^ produced a pronounced decrease in fluorescence intensity (Figure 4a). This behavior is consistent with the selectivity observed in solution, indicating that the N,S-CQDs retain their selective fluorescence response toward Hg^2+^ after immobilization on filter paper. The response of the paper-based sensor to different Hg^2+^ concentrations was further investigated. As shown in Figure 4b, the relative fluorescence intensity I/I_0_ gradually decreases with increasing Hg^2+^ concentration and shows a linear dependence within the tested concentration range:

*I/I*_0_ = 0.9993 − 2.8882*C_Hg_*^2+^

The coefficient of determination was *R*^2^ = 0.9921. Here C_Hg2+_ represents the Hg^2+^ concentration in the treatment solution applied to the paper surface, expressed in mM. The limit of detection was 6.23 μM, indicating a concentration-dependent analytical response toward Hg^2+^ in the paper-based format. For clarity, the calibration equations, linear concentration ranges, coefficients of determination (R^2^), and limits of detection (LODs) for the solution-phase and paper-based sensing systems are summarized in Table 1.

Figure 4c further presents fluorescence photographs of the paper-based sensor under UV irradiation after treatment with different metal ions. The blank and most metal ion-treated samples retain pronounced blue fluorescence, whereas the Hg^2+^-treated area shows substantially weaker fluorescence. These results demonstrate that immobilization of the N,S-CQDs on filter paper preserves the selective response toward Hg^2+^ and enables visual discrimination based on fluorescence changes, providing a basis for portable paper-based detection.

To evaluate the reversibility and reusability of the N,S-CQD paper-based sensor, alternating Hg^2+^/EDTA treatments were performed, as shown in Figure S4. The fluorescence intensity decreased markedly after Hg^2+^ treatment and was subsequently restored following EDTA treatment. This recovery can be attributed mainly to the strong complexation ability of EDTA toward Hg^2+^, which promotes competitive binding of Hg^2+^ and weakens its interactions with the surface functional groups of the N,S-CQDs [28,59]. Consequently, the Hg^2+^-induced perturbation of the surface-related emissive states may be alleviated, allowing partial restoration of the fluorescence emission. This interpretation is also consistent with the changes in the UV-Vis absorption characteristics observed after EDTA addition (Figure 3d). After three alternating Hg^2+^/EDTA treatments, the paper-based sensor maintained a distinct fluorescence quenching-recovery response, although a slight decrease in the recovered fluorescence intensity was observed. These results support the partially reversible nature of the Hg^2+^-N,S-CQD interactions and demonstrate the potential of the paper-based sensor for reusable Hg^2+^ detection. This interpretation is supported by the reversible fluorescence response and relevant literature, although the specific interfacial coordination processes were not directly characterized.

To further evaluate the analytical performance of the developed N,S-CQDs, a comparison with recently reported fluorescent sensors for Hg^2+^ detection is presented in Table S3 [4,27,46,58,60,61,62,63,64]. The solution-phase N,S-CQDs exhibit an LOD of 4.2 nM, which is lower than those of several reported carbon dot-based fluorescent sensors, including N-CDs (7.1 nM), N,S-CDs/AuNCs (18 nM), and B,N,S-CDs (72 nM), although more sensitive systems have also been reported [46]. Furthermore, the N,S-CQDs exhibit a relatively high fluorescence quantum yield of 67.25% and are synthesized using renewable bagasse-derived xylan as the carbon precursor. Although the paper-based sensor exhibits a higher LOD (6.23 μM) than some reported paper-based fluorescent sensors, it retains selective Hg^2+^ detection and reversible fluorescence responses following EDTA treatment. These results highlight the combination of renewable biomass utilization, sensitive solution-phase detection, and reusable paper-based sensing achieved in this work.

### 3.5. Application of N,S-CQDs as Fluorescent Ink

**Figure 5:**
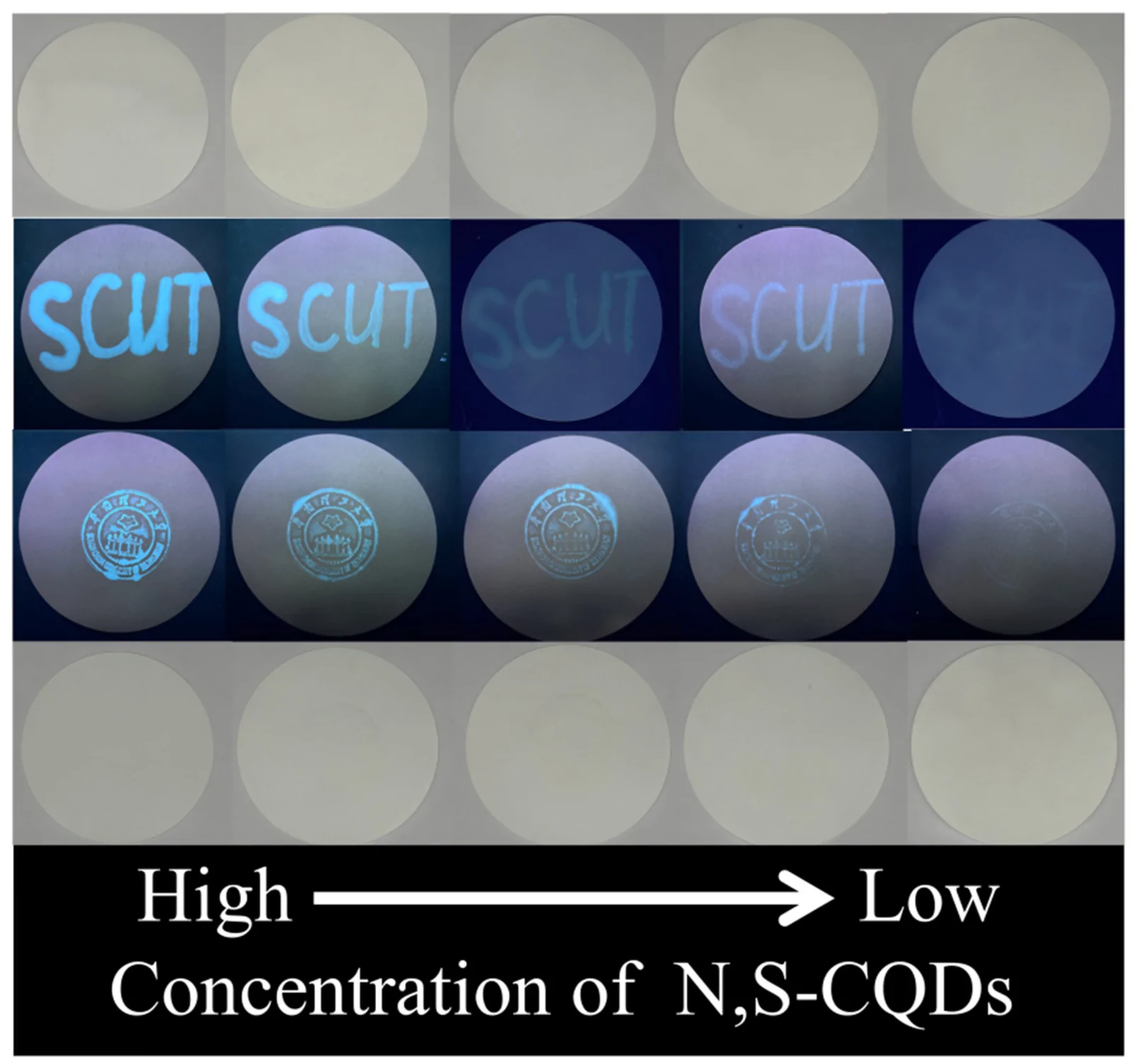
Written text and stamped patterns produced using N,S-CQD fluorescent inks with different relative concentrations under visible light and UV irradiation.

To further evaluate the compatibility of the N,S-CQDs with paper-based processing, dispersions with different relative concentrations were directly used as fluorescent inks to produce text and patterns on filter paper through writing and stamp printing, as shown in Figure 5. The relative concentration of the N,S-CQDs gradually decreases from left to right. Under visible light, the text and patterns are barely distinguishable, whereas under UV irradiation they display clear blue fluorescence. As the relative N,S-CQD concentration decreases, both the fluorescence intensity and visibility of the written and stamped patterns gradually decrease, indicating that the fluorescence readout on paper depends on the amount of N,S-CQDs deposited on the substrate. These results further demonstrate that the N,S-CQDs retain effective solid-state fluorescence on paper and can be directly processed for fluorescent writing and pattern printing [65,66].

## 4. Conclusions

N,S-CQDs were prepared through one-step hydrothermal co-carbonization using bagasse-derived xylan and L-cysteine as precursors. The resulting carbon dots exhibited a mean particle diameter of 3.25 ± 0.04 nm, with optimal excitation and maximum emission wavelengths of 330 and 420 nm, respectively. Under the tested conditions, the N,S-CQDs exhibited a stronger fluorescence quenching response toward Hg^2+^ than toward the other individually tested metal ions, with a detection limit of 4.2 nM in solution. After immobilization on filter paper, the resulting paper-based sensor retained a visually observable, concentration-dependent fluorescence response toward Hg^2+^, with a detection limit of 6.23 μM, and exhibited reversible fluorescence quenching and recovery during repeated Hg^2+^/EDTA treatments. Overall, this work extends the valorization of renewable xylan to functional fluorescent carbon nanomaterials and demonstrates the feasibility of using these materials for Hg^2+^ fluorescence sensing and reusable paper-based detection.

## Figures and Tables

**Table 1:** Calibration parameters and analytical performance of N,S-CQDs for Hg^2+^ detection in solution-phase and paper-based sensing systems.

Detection System	Linear Concentration Range	Calibration Equation	R^2^	LOD
Solution-phase sensing	0–1.0 μM	I/I_0_ = −0.9233C + 0.9418	0.9771	4.2 nM
Paper-based sensing	0.5–9 μM	I/I_0_ = −2.8882C + 0.9993	0.9921	6.23 μM

## Data Availability

Data will be made available on request.

## Supplementary Materials

The following supporting information can be downloaded at: https://www.mdpi.com/article/10.3390/polym18212753/s1. Figure S1. Schematic illustration of the synthesis and applications of xylan-derived N,S-CQDs; Figure S2. (a) XPS survey spectrum and high-resolution (b) C 1s, (c) O 1s, (d) N 1s, and (e) S 2p spectra of N,S-CQDs; Figure S3. (a) Fluorescence intensity as a function of time; (b) fluorescence intensity at different pH values; (c) fluorescence decay curve and biexponential fit; and (d) photographs of N,S-CQD dispersions under UV irradiation; Figure S4. Reversible fluorescence response and reusability of the N,S-CQD paper-based sensor toward Hg^2+^; Table S1. Comparison of characteristic FT-IR absorption bands of xylan and N,S-CQDs; Table S2. Fluorescence lifetime and quantum yield of N, S-CQDs; Table S3. Comparison of the analytical performance and practical characteristics of the proposed N,S-CQDs with recently reported fluorescent sensors for Hg^2+^ detection.
