# Supplementary material for: Xylan-Derived N,S-Codoped Carbon Dots for Hg2+ Detection and Reusable Paper-Based Sensing
Source: Polymers (Basel). 2026 Nov 5;18(21):2753. doi: 10.3390/polym18212753 (PMC13765740; doi:10.3390/polym18212753)
Supplement: Supplementary file 1 [file polymers-18-02753-s001.zip › polymers-4636087-supplementary.pdf]

Supporting Information

Xylan-Derived N,S-Codoped Carbon Dots for Hg^{2+} Detection and Reusable Paper-Based Sensing

Yunqin Qi [1,2], Zhouyang Xiang [2], Qingyi Lin [2] and Zhenhua Hu [1,*]

1 School of Food Engineering, Ludong University, Yantai 264025, China
2 State Key Laboratory of Advanced Papermaking & Paper-Based Materials, South China University of Technology, Guangzhou 510640, China
* Correspondence: zhenhuahu@ldu.edu.cn

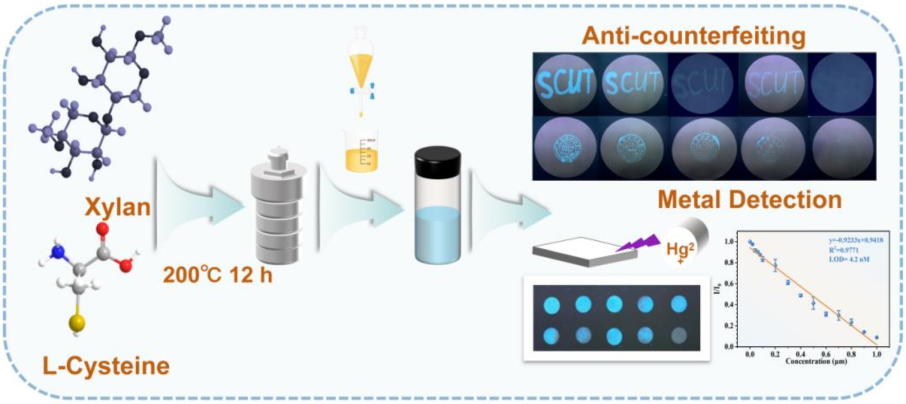

Figure S1. Schematic illustration of the synthesis and applications of xylan-derived N,S-CQDs.

Table S1. Comparison of characteristic FT-IR absorption bands of xylan and N,S-CQDs.

Xylan (cm^{-1})	N,S-CQDs (cm^{-1})	Vibrational assignment	Associated structure
~3442	—	O–H stretching	Hydroxyl groups in xylan
—	~3442	O–H/N–H stretching	Surface functional groups
1000–1170	—	C–O stretching	Polysaccharide structure
1000–1170	—	C–O–C stretching	Glycosidic linkages
~898	—	β-Glycosidic linkage vibrations	Xylan backbone
—	~1706	C=O stretching	Carbonyl-containing groups
—	~1626	C=C stretching and overlapping vibrations	Conjugated carbon structures
—	~1423	C–N/C–O and related vibrations	Surface functional groups
—	~1327	C–N/C–O and related vibrations	Surface functional groups
—	~2600 (weak)	Possible S–H stretching	Potential sulfur-containing groups

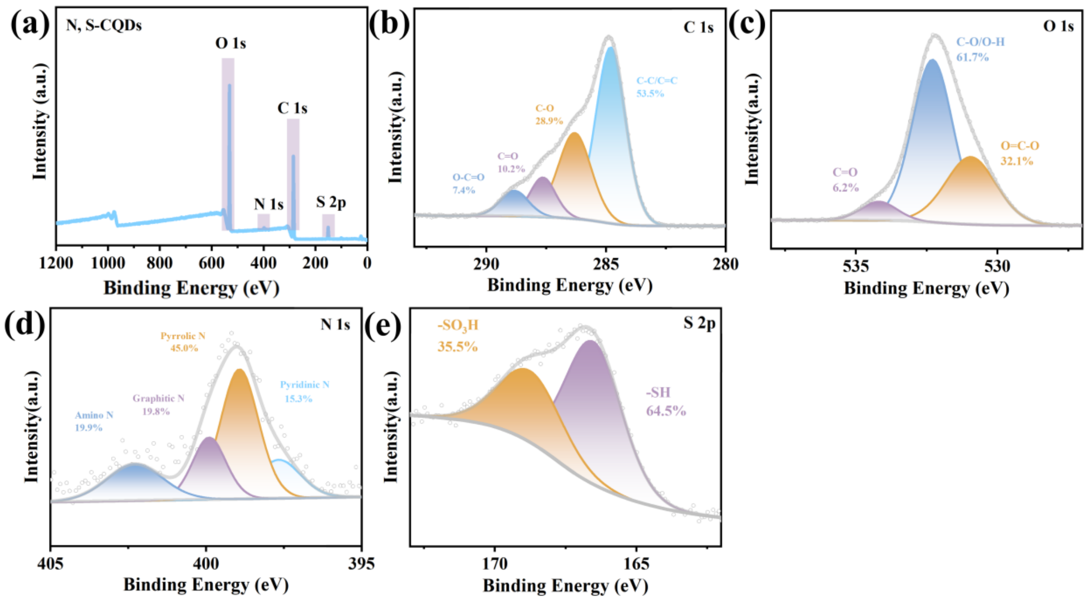

Figure S2. (a) XPS survey spectrum and high-resolution (b) C 1s, (c) O 1s, (d) N 1s, and (e) S 2p spectra of N,S-CQDs.

Table S2. Fluorescence lifetime and quantum yield of N, S-CQDs.

Sample	τ_1 (ns)	τ_2 (ns)	A_1 (%)	A_2 (%)	$\bar{\tau}$ (ns)	Φ (%)
**N, S-CQDs**	1.43	7.71	59.77	40.23	6.35	67.25

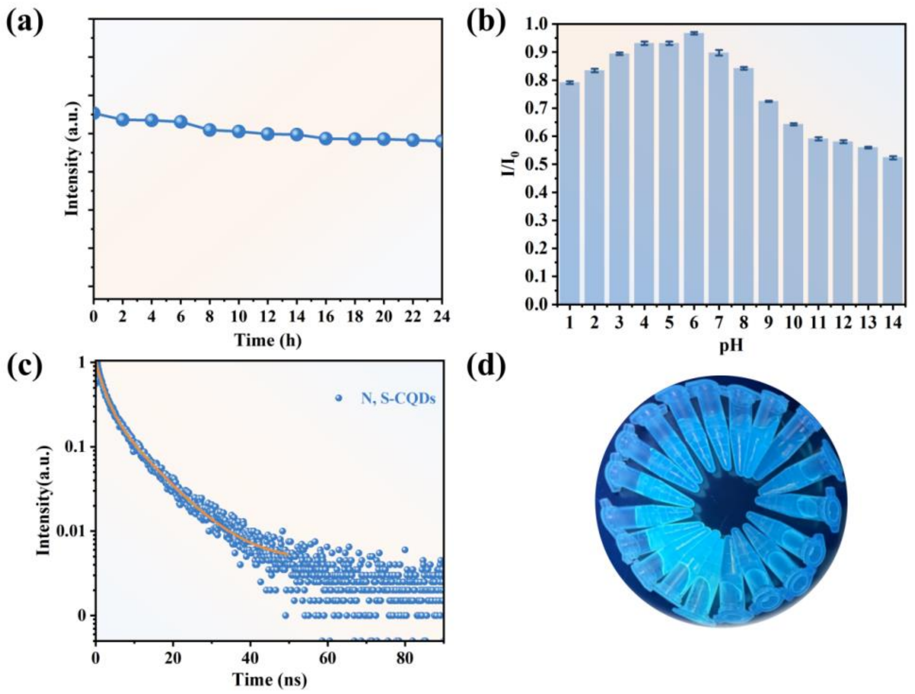

Figure S3. (a) Fluorescence intensity as a function of time; (b) fluorescence intensity at different pH values; (c) fluorescence decay curve and biexponential fit; and (d) photographs of N,S-CQD dispersions under UV irradiation.

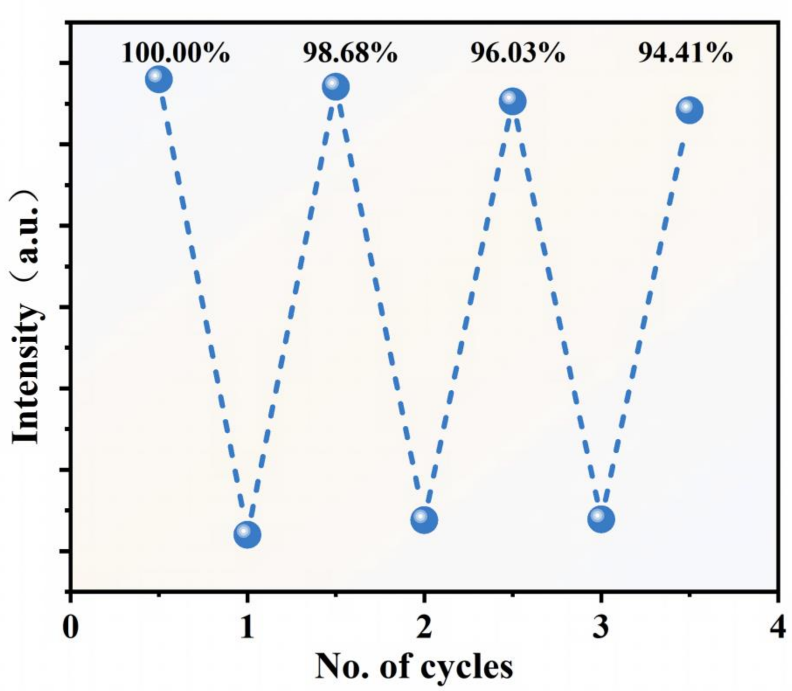

Figure S4. Reversible fluorescence response and reusability of the N,S-CQD paper-based sensor toward Hg^{2+}.

Table S3. Comparison of the analytical performance and practical characteristics of the proposed N,S-CQDs with recently reported fluorescent sensors for Hg^{2+} detection.

Fluorescent sensing material	Carbon source / precursor	Detection mode	Linear range (μM)	LOD (nM)	QY (%)	Ref.
N-CDs	Nitrogen-doped, amide-functionalized CDs	Fluorescence quenching; microfluidic platform	0.007–120	7.1	53.33	[1]
N,S-CDs/AuNCs	N,S-codoped CDs and Au nanoclusters	Ratiometric fluorescence; smartphone-assisted	0.05–3.0; 3.0–7.0	18	NR	[2]
B,N,S-CDs	Boron-, nitrogen-, and sulfur-codoped CDs	Solution-phase fluorescence quenching	NR	72	67.6	[3]
GCDs	*o*-Phenylenediamine and ethylenediamine	Solution-phase fluorescence quenching	0–100	300	23.1	[4]
N-CDs	Nitrogen-doped CDs	Solution-phase fluorescence quenching	0–50	0.28 25	35	[5]
Rice-starch-derived CDs	Rice starch; methimazole functionalization	Paper-based; fluorescence image analysis	0.5–45	230	NR	[7]
N,S-CDs@g-C_3N_4	N,S-codoped CDs/graphitic carbon nitride composite	Solution-phase fluorescence quenching	1.0–7.2	421	NR	[8]
N,S-CQDs	Waste lemon peel and glutathione	Solution-phase fluorescence quenching	0.0176–20	17.6	15.2	[9]
Xylan-derived N,S-CQDs	Bagasse-derived xylan and L-cysteine	Solution-phase fluorescence	0–1.0	4.2	67.25	This work

		quenching				
Xylan-derived N,S-CQDs	Bagasse-derived xylan and L-cysteine	Paper-based fluorescence sensing	0.5-9	6230	NR[c]	This work

(1) R. Ma, L. Chang, L.D. Nghiem, Y. Liu, Q. Wang, Q. Zhao, Q. Hao, Y. Gao, H. Liu, L. Zheng. Ligand-to-Metal Charge Transfer Quenching of Carbon Dots for Highly Selective Hg^{2+} Detection in Microfluidic Devices. ACS Applied Nano Materials. 2024, 7, 22276–22284. DOI: 10.1021/acsanm.4c04700.

(2) J. Zhang, T. Wang, Y. Du, Y. Wu, X. Jiang, H. Zou, Z. Zhang. Smartphone-assisted ratiometric fluorescence sensor based on NS-codoped carbon dots/Au nanoclusters for rapid detection of Hg^{2+}. Microchemical Journal. 2024, 199, 110201. DOI: 10.1016/j.microc.2024.110201.

(3) Y. Ding, Y. Chen, C. Wang. Boron, nitrogen, and sulfur co-doped carbon dots as a fluorescence probe for label-free analysis of Hg^{2+}. Journal of the Chinese Chemical Society. 2024, 71, 1092–1102. DOI: 10.1002/jccs.202400089.

(4) J. Hou, Q. Chen, X. Meng, H. Liu, W. Feng. Synthesis of green fluorescent carbon dots and their application in mercury ion detection. RSC Advances. 2024, 14, 36273–36280. DOI: 10.1039/D4RA06635A.

(5) Y. Mei, S. Yang, M. Su, J. Xie, S. Huang, Z. Jiang, Y. Huang. Nitrogen-doped carbon dots for ultrasensitive detection of Hg^{2+} in traditional Chinese medicine and bioimaging applications. Analytical Methods. 2025, 17, 5400–5411. DOI: 10.1039/D5AY00704F.

(6) Y. Gao, L. Zhou, Z. Li, P. Sun, Q. Dai, R. Xie, F. Chai. The tunable synthesis of orange emissive carbon dots for sensitive and selective detection of Hg^{2+} via portable paper sensor. Journal of Environmental Chemical Engineering. 2024, 12, 111834. DOI: 10.1016/j.jece.2023.111834.

(7) M.V. Maia, W.T. Suarez, V.B. dos Santos, S.C.B. de Oliveira, J.P.B. de Almeida. A novel approach to Hg^{2+} determination in water samples using carbon dots based on paper and fluorescence digital image analysis. Journal of Chemical Technology & Biotechnology. 2024, 99, 1157–1164. DOI: 10.1002/jctb.7618.

(8) Rajesh, R. Singh, S. Swami, S. Bhogal. Nitrogen and Sulfur Codoped Carbon Dots Graphitic Carbon Nitride Composites for Fluorescence Sensing of Mercury Ions. Luminescence. 2025, 40, e70398. DOI: 10.1002/bio.70398.

(9) Y. Pan, S. Guo, T. Wang, J. Lin, Z. Jia, F. Xu, R. Huang, H. Wang, C. Bai, J. Chen. DMF-mediated synthesis of N,S-co-doped carbon quantum dots and their fluorescence quenching mechanism toward Hg^{2+} via surface complexation. RSC Advances. 2026, 16, 2652–2662. DOI: 10.1039/D5RA08448B.
